# Pharmacogenomics biomarkers for personalized methadone maintenance treatment: The mechanism and its potential use

**DOI:** 10.17305/bjbms.2020.4897

**Published:** 2021-04

**Authors:** Fitri Fareez Ramli

**Affiliations:** Department of Pharmacology, Faculty of Medicine, Universiti Kebangsaan Malaysia, Cheras, Kuala Lumpur, Malaysia

**Keywords:** Methadone, pharmacokinetics, pharmacogenomics, personalized medicine, *ABCB1*, *CYP2B6*, *CYP2C9*, *CYP2C19*, *CYP3A4*, *CYP2D6*

## Abstract

Methadone has a wide pharmacokinetic interindividual variability, resulting in unpredicted treatment response. Pharmacogenomic biomarkers seem promising for personalized methadone maintenance treatment. The evidence supports the use of *ABCB1* single-nucleotide polymorphism (SNP) 1236C>T with genotypes C/T or C/C (Jewish) and haplotypes AGCTT carrier, AGCGC heterozygote, or non-carrier (Caucasian), which have a predicted lower methadone dose requirement. In contrast, *ABCB1* SNP 1236C>T with genotype T/T (Jewish); haplotypes AGCGC homozygote, AGCTT non-carrier (Caucasian), and *ABCB1* 3435C>T variant carrier; and haplotypes CGT, TTC, and TGT (Han Chinese) have a predicted higher methadone dose. For methadone plasma levels, *ABCB1* diplotype non-CGC/TTT (Malay) predicted lower, and diplotype CGC/TTT (Malay), 3435C>T allelic carrier, haplotypes (CGT, TTC, TGT) (Han Chinese) predicted higher methadone levels. In terms of metabolism biomarkers, a lower methadone requirement was related to carriers of *CYP2B6* genotypes *4(G/G) and *9(T/T) among Jewish patients, CYP2B6*9 genotype (T/T) and haplotypes (TA/TG); and *CYP2C19* (*2/*2,*2/*3, and *3/*3; Han Chinese). Higher methadone dose was observed in CYP2C19*1 allelic carriers (Han Chinese) and *CYP2D6* ultrarapid metabolizer (Caucasian). Lower methadone levels were reported in *CYP2B6* SNPs, haplotypes TTT, and AGATAA (Han Chinese), *CYP2C19* genotype *1/*1 (Han Chinese), allelic carrier *1xN (Caucasian), and *CYP3A4* genotype *1/*1 (Caucasian). Carriers of *CYP2B6* genotype *6/*6 (Caucasian), *CYP2B6* haplotypes ATGCAG and ATGCTG (Han Chinese), and *CYP3A4* genotype *1/*1B (Caucasian) had predicted higher methadone plasma levels. Specific pharmacokinetics biomarkers have potential uses for personalized methadone treatment in specific populations.

## INTRODUCTION

Methadone maintenance treatment (MMT) is a program widely available to treat opioid use disorder [1]. MMT is proven in reducing opioid and other illicit drug use, human immunodeficiency virus (HIV) risk-taking behavior, and crime. Significant improvements in various aspects of life, such as health, social functioning, and quality of life, are other positive outcomes of MMT [2]. Administration of the optimal methadone dose is essential to achieve effective treatment. Inadequate dosing may exacerbate withdrawal symptoms, resulting in illicit drug use and other risk-taking behavior [3]. Hence, what dose is required for optimal MMT? No dose range is specified in clinical guidelines. Reports from numerous studies have revealed a wide range of effective methadone dosing, with the mean effective dose ranging from 54.7 to 140.0 mg/day [4-11]. The range of plasma concentration of methadone may also vary from as low as 100 ng/mL to as high as 1220 ng/mL [7]. Furthermore, a meta-analysis study reported better retention rates in flexible doses than fixed methadone doses in MMT patients [12]. This result indicates that individualized methadone dosing correlates to better MMT outcomes.

The broad range of methadone dosage may be attributed to genetic polymorphisms. Numerous studies have shown the potential contribution of genetic polymorphisms to the variability of both methadone dose and concentration [4-7,10,11,13-18]. Recent advances in MMT reveal the potential use of pharmacogenomics markers for individualized methadone treatment. Numerous studies have found a significant association between pharmacokinetic genetic markers and methadone dose, concentration, and treatment outcomes such as withdrawal symptoms and adverse reactions [10,13].

Methadone is a synthetic opioid that is available in a racemic mixture of *(R)-* and *(S)-*enantiomers in equal proportion. *(R)-*methadone has been reported to account for most opioid effects, including the primary analgesic effect [4,13]. The presence of adequate methadone levels in the central nervous system is essential for optimal pharmacological effects. High levels of methadone may cause cardiac arrhythmia [19], respiratory arrest, and death [20]. Insufficient dosing leads to withdrawal symptoms. Wide interindividual variability of methadone dosage is a challenge in achieving optimal methadone treatment. Various factors have been reported as correlates to this variability such as age, diseases, comedication, and genetic polymorphisms [14]. Pharmacokinetic factors play an essential role in delivering methadone from the site of absorption to the site of action. This process includes absorption, distribution, metabolism, and excretion. P-glycoprotein (P-gp) plays a crucial role in absorption, distribution, and elimination [21,22]. In terms of metabolism, cytochrome P450 (CYP) is significant in the inactivation of methadone. Numerous studies have reported the association between genetic polymorphisms of both P-gp and CYPs and methadone treatment factors [4-7,10,11,13-18]. This review aims to elaborate on the mechanism of P-gp and CYP genetic polymorphisms that contributes to the variability of methadone pharmacokinetics. Moreover, it summarizes the potential use of genetic biomarkers involved in pharmacokinetics for personalized methadone treatment in terms of dose and plasma concentration levels of methadone.

## P-GP

**FIGURE 1:**
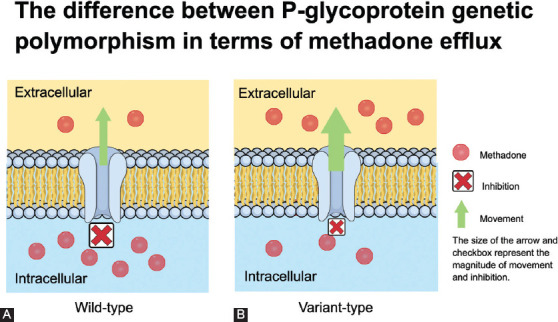
The differences between wild-type and variant-type of P-gp in methadone efflux. (A) Note that methadone causes more potent inhibition towards P-gp in the wild-type genetic polymorphism, resulting in less methadone being transported out. (B) In contrast, methadone causes less potent inhibition towards P-gp in the variant-type genetic polymorphism, resulting in more methadone being transported out. P-gp: P-glycoprotein.

P-gp is an efflux pump, which actively transports xenobiotics out of the intracellular compartment to prevent toxic cellular effects [21]. It belongs to the adenosine triphosphate (ATP)-binding cassette (ABC) B (ABCB) subfamily [22]. P-gp is expressed in numerous tissues such as intestines and brain [23]. Both *(R)-* and *(S)*-methadone are P-gp substrates [24-26]. Preclinical studies reported the importance of P-gp in the blood–brain barrier. P-gp knockout mice had a higher methadone concentration in the brain compared to wild-type mice [24-26]. An *in vitro* study reported the ability of methadone to inhibit both the wild-type and variant-type human P-gp; the former had a more potent effect than the latter (Figure 1) [27]. Methadone binding to P-gp results in conformation, which inhibits ATP hydrolysis and transport [28]. The inhibition of P-gp by methadone results in less methadone requirement as more methadone is available at the site of action. On the other hand, methadone at therapeutic doses can inhibit P-gp ATPase activity stimulated by other drugs, resulting in drug–drug interaction [27,29]. Numerous *ABCB1* genetic polymorphisms have been found to affect methadone pharmacokinetics, resulting in a wide interindividual variability [23].

### Methadone dose

Multiple studies have reported the association between the *ABCB1* genetic variants and methadone dose (Table 1). In a study of a Jewish population, Levran et al. [16] found a significant difference in genotype frequencies of the *ABCB1* single-nucleotide polymorphism (SNP) 1236C>T between lower- and higher-dose groups. Homozygotes with T allele had more than 6 times risk to require more than 150 mg/day stabilizing methadone dose than those who were heterozygotes or homozygotes for C allele (Table 2). Multi-locus genotype pattern analysis initially found a significant association between genotype distribution (a combination of 1236C>T, 2677G>T/A, and 3435C>T) and methadone dose, but this became negligible in multiple testing analysis [16]. Coller et al. [14] reported that two haplotypes of *ABCB1* (AGCGC and AGCTT) were associated with methadone dose. These haplotypes were formed from 12 unique combinations from five common SNPs in the Caucasian population: 61A>G, 1199G>A, 1236C>T, 2677G>T, and 3435C>T. Possession of two copies of the AGCGC haplotypes was associated with a higher methadone dose compared to having a single copy or none. In contrast, the AGCTT haplotype carrier was associated with a significantly lower methadone dose requirement. Hung et al. [30] reported a significant association between *ABCB1* 3435 C>T allelic variant carriers and non-carriers in terms of methadone dose in a Han Chinese population. A higher dose was required in carriers (homozygotes>heterozygotes) than non-carriers [30]. Moreover, *ABCB1* haplotypes of CGT, TTC, and TGT, a combination of 1236C>T, 2677G>T/A, and 3435C>T had a significantly higher methadone requirement in the Han Chinese population [30].

**TABLE 1:**
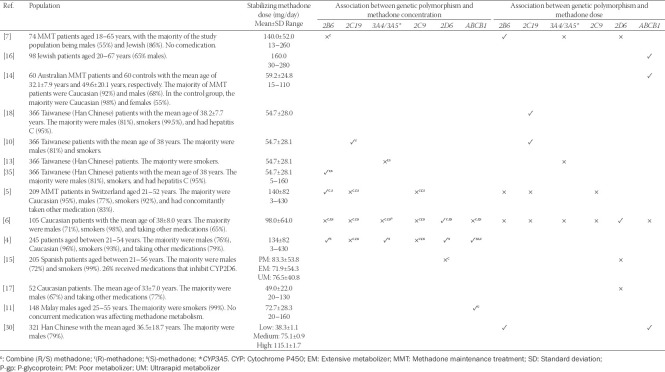
The associations of genetic polymorphism and methadone concentration or methadone dose in different populations

**TABLE 2:**
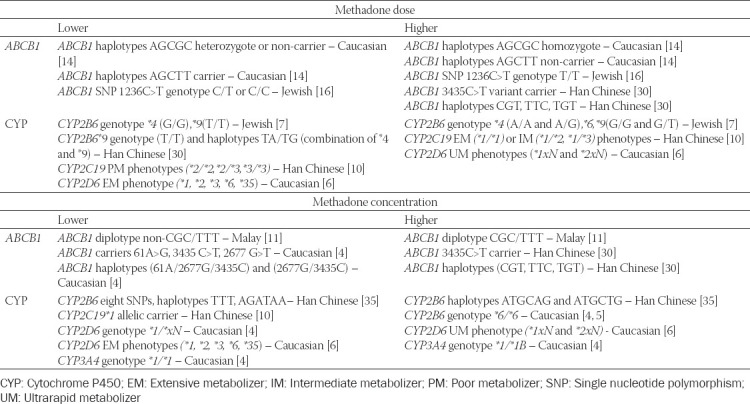
Potential genetic polymorphisms for the prediction of methadone dose and concentration

In contrast, Fonseca et al. [6] reported a negligible difference in terms of methadone dose between the *ABCB1* phenotypes of extensive metabolizer (EM), intermediate metabolizer (IM), and poor metabolizer (PM) in a Caucasian population. Another study in the Caucasian population also reported no significant association between responders’ status and *ABCB1* genotypes [4].

A comparison of methadone dose was different between studies. Two studies with significant findings predefined the methadone dose group into (1) low (≤150 mg) and high dose (>150 mg) [16] and (2) low (<55 mg), medium (55–99 mg), and high dose (100–150 mg) [30]. Another study with significant findings compared the difference of methadone dose between haplotypes [14]. Two studies found negligible associations between phenotype [6] and responder status: low-dose responder (40–80 mg), high-dose responder (≥120 mg), or non-responder (≥120 mg) [4].

### Methadone concentration

In a Malay population, Zahari et al. [11] reported that *ABCB1* CGC/TTT diplotype (a combination of 1236C>T, 2677G>T/A, and 3435C>T) was significantly associated with higher *(R,S)-*methadone plasma levels compared to those who did not possess this diplotype. Other diplotypes, haplotypes, genotypes, or allelic variants had insignificant contributions towards methadone plasma levels. Similarly, a Caucasian population study found a similar negligible difference in *(R*)-, (*S*)-, and (*R,S)*-methadone plasma levels between three phenotypes of *ABCB1* 3435 C>T polymorphism [6]. However, it did not analyze the difference in terms of diplotype, as did Zahari et al. [11]. In contrast, another study in the Caucasian population reported a significant association between *ABCB1* 61A>G and 3435C>T, and trough (*R*)-, (*S*)-, and (*R,S*)-methadone plasma levels. Lower methadone levels were observed in carriers of allelic variants *ABCB1* 61A>G (A/G or G/G genotypes) and *ABCB1* 3435C>T (C/T or T/T genotypes). Higher methadone plasma concentrations were observed in carriers of *ABCB1* haplotypes 61A/2677G/3435C and 2677G/3435C. No stereoselectivity in P-gp transport for methadone was reported [4].

## CYPS

**FIGURE 2:**
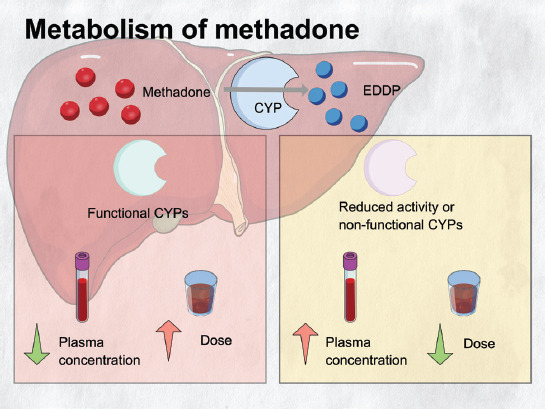
This figure shows the metabolism of methadone to EDDP by CYP enzymes. This figure also shows the difference between functional and reduced activity or non-functional CYPs in terms of methadone plasma concentration and dose. In the presence of functional enzymes, more methadone can be metabolized, resulting in reduced plasma concentration and perhaps a higher methadone dose. In contrast, reduced enzyme activity or non-functional enzyme results in higher methadone plasma concentration and perhaps a lower dose of methadone EDDP: 2-ethylidene-1,5-dimethyl-3,3-diphenylpyrrolidine; CYP: Cytochrome P450.

Methadone metabolism mainly occurs in the liver [1]. CYP in the Phase I reaction is essential for the N-demethylation of methadone, producing the inactive metabolite 2-ethylidene-1,5-dimethyl-3,3-diphenylpyrrolidine (EDDP). The primary CYPs involved in methadone metabolism are cytochrome P450 2B6 (CYP2B6), CYP2C19, and CYP3A4. CYP3A4 shows no preference towards any methadone isomers, and CYP2C19 has selectivity towards the *(R)-*methadone isomer [31]. Inhibition of CYP2B6 significantly reduces the metabolism of both isomers with a three-fold increase in plasma *(S)-*methadone over the other isomer [32]. This shows the stereoselectivity of CYP2B6 towards (*S*)-methadone. Other methadone-related CYPs include CYP2D6 and CYP2C9. The number of functional alleles of a CYP genotype determines the individual phenotype in the metabolism rate. CYP phenotypes include PM, IM, EM, and ultrarapid metabolizer (UM). In general, in the presence of functional enzymes, more methadone can be metabolized, resulting in reduced plasma concentration and perhaps a higher methadone dose. In contrast, reduced enzyme activity or enzyme non-functionality results in higher methadone plasma concentration and perhaps a lower dose of methadone (Figure 2). Numerous studies have reported the contribution of CYP genetic polymorphisms to withdrawal symptoms, adverse effects, and methadone dose [13,18].

### CYP2B6

CYP2B6 is the primary enzyme responsible for methadone metabolism. CYP2B6 produces the highest EDDP metabolite compared to other CYP families and it showed stereoselectivity towards *(S)-*methadone in a preclinical study [31]. A clinical study proved CYP2B6 as the primary driver of methadone inactivation of both *(R)-* and *(S)-*enantiomers with stereoselectivity towards the latter enantiomer [32]. *(S)-*methadone is associated with adverse effects related to methadone. Ansermot et al. [33] reported a significant QTc reduction in patients receiving a substitution of *(R)-*methadone, who were previously on *(R,S)-*methadone. Moreover, a study of methadone-related death found significantly higher methadone plasma levels in CYP2B6*6 allele carriers than CYP2B6*1 and *4. This result indicates the role of *CYP2B6* in methadone metabolism with the carriers of the non-functional CYP2B6*6 representing the poor phenotype group [34].

#### Methadone dose

Levran et al. [7] reported that a significantly lower methadone dose (<100 mg/day) was required to stabilize MMT patients who were homozygotes for the variant alleles of CYP2B6*4 and CYP2B6*9 compared to those who were heterozygotes and non-carriers. The result was not attenuated even after controlling for other confounding factors such as age, sex, and the *ABCB1* 1236T/T genotypes. These variant alleles corresponded to CYP2B6*6. The associations between the additional SNPs of *CYP2B6* and methadone dose were not significant [7]. In the Han Chinese population, a similar finding was observed in CYP2B6*9 carriers, who required a significantly lower dose than non-carriers [30]. Moreover, haplotype analysis in this population reported haplotypes combining allelic variants *4 (516G>T) and *9 (785A>G) with TA or TG, who had a significantly lower methadone dose requirement compared to non-carriers [30].

Reports in the Caucasian population have found a negligible difference between *CYP2B6* genotypes in methadone dose [5,6]. The contradictory findings might be due to differences in inclusion and exclusion criteria, ethnicity, and analysis. Levran et al. [7] had more stringent criteria that only included subjects using no concomitant medications known to affect methadone metabolism. In contrast, Crettol et al. [5] and Fonseca et al. [6] did not exclude patients using such concurrent medications, with the earlier study [5] only excluding the comedication in the high-dose non-responder group. Methadone dose group classifications were different between studies based on genotypes [7], phenotypes [6], and predefined dose groups: low-dose responders (40–80 mg/day), high-dose responders (≥120 mg/day), and high-dose non-responders (≥120 mg/day) [5]. The analysis of the association between CYP2B6*6 and methadone dose was different between two studies as one analyzed CYP2B6*4 and CYP2B6*9 genotypes separately. These alleles had been shown to have strong linkage disequilibrium and could represent CYP2D6*6 [7]. Other studies have analyzed the methadone dose between CYP2B6*6 genotypes [5,6].

#### Methadone concentration

In terms of methadone concentration, Crettol et al. [5] found significantly higher trough and peak plasma levels of *(S)-* and *(R,S)-*methadone in homozygous carriers of the CYP2B6*6 allele variant than in heterozygotes and non-carriers in a Swiss MMT population. A similar finding was reported by Crettol et al. [4] in another study of the association between trough and peak *(S)-*methadone plasma levels and CYP2B6*6/*6 genotypes. In a Han Chinese population, a similar pattern was observed in carriers of the CYP2B6*6 (rs2279343 and rs3745274) allelic variants. Other SNPs (rs10500282, rs10403955, rs2279345, rs1038376, and rs707265) were also found to be significantly associated with *(S)-*methadone plasma levels. The SNP rs8100458 was significantly associated with trough *(R)-*methadone plasma levels. Moreover, TTT (a combination of rs8100458-rs10500282-rs10403955) and AGATAA (a combination of rs2279342-rs3745274-rs2279343-rs2279345-rs1038376-rs707265) haplotypes were associated with lower *(S)-*methadone plasma levels. ATGCAG and ATGCTG haplotypes were associated with higher *(S)-*methadone plasma levels [35].

In a Caucasian population, the means of both the trough *(S)-* and *(RS)-*methadone plasma levels were higher in homozygous carriers of CYP2B6*6. However, the result did not reach significant levels [6]. In a Jewish population, both SNPs of CYP2B6*4 (SNP785A>G, rs2279343) and CYP2B6*9 (SNP516G>T, rs3745274) were also found not significantly associated with trough *(R/S)-*methadone plasma levels [7]. The effect on specific *(R)-* and *(S)-*methadone plasma levels was not evaluated [7]. The small study sample size is one of the factors that contributed to the negligible association.

### CYP2C19

*CYP2C19* is another enzyme necessary for the metabolism of methadone. An *in vitro* study reported a similar production of EDDP by CYP3A4 with stereoselectivity towards *(R)-*methadone enantiomer [31,36]. In clinical studies, the *(R)-*methadone stereoselectivity of CYP2C19 has been conflicting [5,6,10].

#### Methadone dose

Studies in a Han Chinese population reported a significant association between methadone dose and *CYP2C19* phenotypes among the Han Chinese population [10,18]. Both EM (*1/*1) and IM (*1/*2, *1/*3) groups had significantly higher methadone dose than PM (*2/*2, *2/*3, *3/*3) group [10]. The significant levels were enhanced with the inclusion of allele types in either *CYP2B6* or *CYP3A4*, as well as the allelic combination of those CYPs included in the analysis, which allowed the formation of 6–12 different methadone dose ranges [18]. However, the authors did not elaborate more on the proposed classification [18]. Gene-gene interaction analysis found a negligible association between these major CYPs in methadone metabolism [18].

In contrast, Crettol et al. [5] found no significant difference in *CYP2C19* genotype distribution between responders and non-responders, as well as between low-dose and high-dose groups among a Caucasian population in Switzerland. Similarly, another study in a Caucasian population in Spain reported negligible methadone dose difference between EM (*1/*1), IM (*1/*2), and PM (*2/*2) phenotypes of *CYP2C19* [6]. The contradictory findings might be attributed to the inclusion of comedication known to affect methadone metabolism by *CYP2C19* [5,6]. Wang et al. [10] performed a further analysis that removed participants with comedication known to affect *CYP2C19* metabolism, resulting in increased significant levels.

#### Methadone concentration

Wang et al. [10] reported a significant association between *(R)-*methadone plasma levels and *CYP2C19* among Han Chinese with negative urine morphine. Patients with the predicted phenotype of EM (*1/*1) reported a significantly lower trough *(R)-*methadone plasma levels than PM, but a negligible difference was found in terms of *(S)*-methadone plasma levels. The SNPs CYP2C19*2 and *3 represented the non-functional alleles while the SNP CYP2C19*1 represented the functional allele. Lack of functional allele variants in the genotype leads to reduced metabolism of *(R)-*methadone, resulting in higher methadone plasma levels [10].

In contrast, Crettol et al. [5], Crettol et al. [4], and Fonseca et al. [6] reported negligible correlations between trough *(R)-, (S)-*, and *(R,S)-*methadone plasma levels and *CYP2C19* genotypes. Stereoselectivity towards *(R)-*methadone was not observed in two studies [5,6], contradicting the reports of other *in vitro* study and *in vivo* study [10,31].

Classification of genotypes, phenotypes, and illicit opioid use may have contributed to the differing findings. Crettol et al. [5] classified phenotypes into two groups: EM (based on genotypes *1/*1, *1/*2, and *1/*3) and PM (based on genotypes *2/*2). Wang et al. [10] divided patients into urine opiate-positive and -negative groups before three further phenotype classifications: EM (based on genotype *1/*1), IM (based on genotypes *1/*2 and *1/*3), and PM (based on genotypes *2/*2, *2/*3, and *3/*3). The genotypes distribution between studies was also different, even between two homogenous populations. The genotype *CYP2C19* *2/*2, for example, had a frequency of 10.7% in the Han Chinese population [10], and 0.95% and 4.8% in the Caucasian population in Spain [6] and Switzerland [5], respectively. The latter two populations consisted of at least 95% Caucasian.

### CYP3A4/5

CYP3A4 and CY3A5 belong to the CYP3A family [37]. An initial finding reported a leading role of CYP3A4 in the metabolism of methadone [38]. A subsequent study found that CYP2B6 is the primary enzyme essential for methadone metabolism [31].

#### Methadone dose

Chen et al. [13] reported no significant association between six SNPs of *CYP3A4* and methadone dose in a Han Chinese population. Similarly, Levran et al. [7] found no significant association between four SNPs of *CYP3A4* and methadone dose in a Jewish population. Despite similar findings in terms of methadone dose in both Han Chinese and Jewish populations, the majority of SNPs included in the final analysis were different, with only one similar SNP (rs2242480). These differences exist due to the differences in the selection method, references, software, methods, and ethnicity. The Caucasian population showed a negligible difference in methadone doses between EM, PM, and very PM phenotypes in terms of *CYP3A5* [6].

#### Methadone concentration

CYP3A activity analysis using midazolam reported higher levels of all forms of methadone at both trough and peak in low CYP3A activity group in a Caucasian population [4]. Crettol et al. [4] reported significantly higher trough and peak *(S)-*methadone plasma levels in the CYP3A4 (*1/*1B) genotype than the CYP3A4(*1/*1) genotype, with an insignificant difference in both trough and peak *(R)-*methadone plasma levels in the initial analysis. However, further analysis found no stereoselectivity towards *(S)-*methadone. Furthermore, no association was found between (*R*)-, (*S*)-, and (*R,S*)-methadone plasma levels at either trough and peak with *CYP3A5* genotypes [4]. Similarly, Fonseca et al. [6] reported an insignificant association between all forms of (*R*)-, (*S*)-, and (*RS)-*methadone plasma levels and *CYP3A5* phenotypes (EM, PM, and very PM). A study in a Han Chinese population reported no significant difference in trough *(R)-* and (*S)-*methadone plasma levels between all selected *CYP3A4* SNPs [13]. No allelic variant of *1B was included in the study because the variant is rare in the study population [13].

### CYP2C9

In an *in vitro* study, CYP2C9 was found to generate a measurable amount of EDDP only at 10 times the concentration at which major CYPs (2B6, 2C19, and 3A4) were able to generate the measurable amount of methadone metabolites [31].

#### Methadone dose

Crettol et al. [5] reported no significant association between *CYP2C9* and methadone dose between low- and high-dose groups or between responder and non-responder groups. A study in another Caucasian population reported a higher methadone requirement in EM than IM and PM phenotypes. However, the differences did not reach significant levels [6].

#### Methadone concentration

The studies in the Caucasian population reported a negligible association between *(R)-, (S)-*, and *(RS)*-methadone plasma levels and *CYP2C9* phenotypes and genotypes [4,5]. In these studies, the difference in methadone dose was compared between PM (based on genotypes *2/*2, *2/*3, and *3/*3) and EM (based on genotypes *1/*1, *1/*2, and *1/*3), and did not reach significance [5]. Fonseca et al. [6] reported similar findings between EM (*1/*1 and *1/*2), IM (*1/*3), and PM (*2,*3) phenotypes among a Caucasian population.

### CYP2D6

The contribution of CYP2D6 to methadone metabolism is minimal in comparison to other CYPs, as reported in the *in vitro* study. Clinical studies indicated a possible interaction of genetic polymorphisms in methadone metabolism [4,6]. Up to 25 genotypes have been detected in the MMT population [6].

#### Methadone dose

Fonseca et al. [6] reported a significant difference between UM and EM *CYP2D6* phenotypes in trough methadone plasma levels in a Caucasian population, with the former group requiring a significantly higher dose. The possession of at least one functional allele (*1,*2,*3,*6, or *35) was classified as EM in this study. UM was defined as those who possessed duplicates of *1 or *2 [6].

In contrast, de los Cobos et al. [15] in a study of a Caucasian population reported no significant association between PM, EM, and UM phenotypes and methadone dose. The study classification of phenotypes was based on the number of functional alleles (*1,*2, *9, and *10) with none, 1–2, and >2 classified as PM, EM, and UM, respectively [15]. Similarly, Shiran et al. [17] reported no significant association between methadone dose and the activity of CYP2D6 among a Caucasian population in the UK. The classification of phenotypes in the study was different, with phenotypes being grouped based on the CYP2D6 activity using the O-demethylation of dextromethorphan. Furthermore, studies in Jewish [7] and Caucasian patients [4] reported no association between *CYP2D6* SNPs and methadone dose.

#### Methadone concentration

All forms of trough methadone *(R, S*, and *R/S)* were significantly higher in UM compared to EM in a study among a Caucasian population in Spain. We expected that plasma methadone levels should be lower in UM than EM because the expected UM activity is higher than EM. The findings might be due to the inhibition of CYP2D6 methadone metabolism because the majority (65%) of the study population had comedication, such as anti-depressants and antiretrovirals [6]. Crettol et al. [4] reported significantly lower trough *(S)-*methadone plasma levels in IM and EM groups than UM (*1/*xN) group and a negligible difference in trough *(R)-*methadone and peak of all forms of methadone plasma levels.

Another study conducted in Spain reported no significant difference in trough *(R/S)*-methadone plasma levels between phenotypes. In that study, patients using concurrent medication known to inhibit CYP2D6 were not excluded from the analysis [15].

The importance of CYP2D6 to methadone pharmacokinetics is limited because it is not the major CYP involved in methadone metabolism [31]. However, the inhibition of this CYP by methadone has a clinical implication in the metabolism of CYP2D6 substrates, such as dextromethorphan [17,39].

## PERSPECTIVE AND CONCLUSION

**FIGURE 3:**
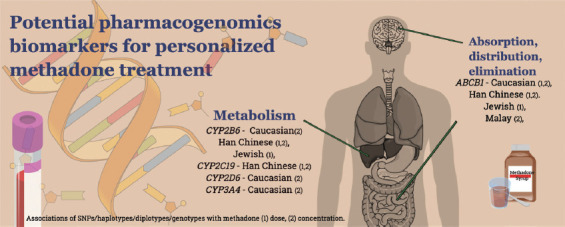
Potential pharmacogenomics biomarkers for personalized methadone treatment in specific populations.

The evidence supports the use of *ABCB1* SNP 1236C>T with genotypes C/T or C/C (Jewish) [16] and haplotypes AGCTT carrier, AGCGC heterozygote, or non-carrier (Caucasian) [14], which have a predicted lower methadone dose requirement. In contrast, *ABCB1* SNP 1236C>T with genotype T/T (Jewish) [16]; haplotypes AGCGC homozygote, AGCTT non-carrier (Caucasian) [14], and *ABCB1* 3435C>T variant carrier; and haplotypes CGT, TTC, and TGT (Han Chinese) [30] have a predicted higher methadone dose. For methadone plasma levels, *ABCB1* diplotype non-CGC/TTT (Malay); *ABCB1* carriers of 61A>G, 3435C>T, and 2677G>T (Caucasian); and *ABCB1* haplotypes of 61A/2677G/3435C and 2677G/3435C in Caucasian [4] predicted lower methadone levels. Diplotype CGC/TTT (Malay) [11]; *ABCB1* 3435C>T allelic carrier (Han Chinese); haplotypes CGT, TTC, and TGT (Han Chinese) [30] predicted higher methadone levels. In terms of metabolism biomarkers, a lower methadone requirement was related to carriers of *CYP2B6* genotypes *4 (G/G) and *9 (T/T) among Jewish patients [7], *CYP2B6* genotype *9 (T/T) and haplotypes (TA/TG) [30]; and *CYP2C19* (*2/*2,*2/*3, and *3/*3; Han Chinese) [10]; and *CYP2D6* EM compared to UM (Caucasian) [6]. Higher methadone dose was observed in carriers of *CYP2B6* genotypes *4 (A/A, A/G) and *9 (G/G and G/T) among Jewish patients [7], CYP2C19*1 allelic carriers (Han Chinese) [10], and *CYP2D6* UM phenotypes (*1xN, *2xN) in Caucasian patients [6]. Lower methadone levels were reported in *CYP2B6* SNPs, haplotypes TTT and AGATAA (Han Chinese) [35], *CYP2C19* genotype *1/*1 (Han Chinese) [10], allelic carrier *1xN [4] and EM phenotypes (Caucasian) [6], and *CYP3A4* genotype *1/*1 (Caucasian) [8]. Carriers of *CYP2B6* genotype *6/*6 (Caucasian) [4,5], CYP2B6 haplotypes ATGCAG and ATGCTG (Han Chinese) [35], *CYP2D6* UM phenotypes (*1xN and *2xN) [6], and *CYP3A4* genotype *1/*1B (Caucasian) [4] had predicted higher methadone plasma levels (Figure 3). Given the small sample sizes in various studies, replication studies with larger sample sizes in similar and different populations are required. Different methodologies hinder a point-by-point comparison between studies. The absence of analysis of the associations between methadone dose and plasma methadone concentration and its impact on specific pharmacogenomic biomarkers in the included studies results in the difficulty in determining direct associations between methadone dose and plasma concentration for specific biomarkers. Because methadone response is related to numerous factors, the analysis should be designed to control as many potential confounding factors as possible, including other pharmacokinetic biomarkers, pharmacodynamic biomarkers, age, sex, and concurrent medication. Artificial intelligence and machine learning are some of the promising tools in treatment outcome prediction. These tools can incorporate pharmacogenomics biomarkers and other datasets such as neuroimaging to produce deep learning algorithms, which can enhance treatment prediction [40].

Specific pharmacokinetics biomarkers have potential uses for personalized methadone treatment in specific populations. The use of pharmacogenomics is essential because methadone has a wide interindividual variability and various possible drug–drug interactions with unpredictable treatment response. More studies are required to validate the use of these biomarkers.

## References

[1] Ramli FF, Shuid AN, Mohamed P, Mohamed R, Mohamed IN (2020). Erectile dysfunction and methadone maintenance therapy. Med Health.

[2] Ali N, Aziz SA, Nordin S, Mi NC, Abdullah N, Paranthaman V (2018). Evaluation of methadone treatment in Malaysia:Findings from the Malaysian methadone treatment outcome study (MyTOS). Subst Use Misuse.

[3] Hayashi K, Ti L, Ayutthaya PP, Suwannawong P, Kaplan K, Small W (2017). Barriers to retention in methadone maintenance therapy among people who inject drugs in Bangkok, Thailand:A mixed-methods study. Harm Reduct.

[4] Crettol S, Déglon JJ, Besson J, Croquette-Krokar M, Hämmig R, Gothuey I (2006). ABCB1 and cytochrome P450 genotypes and phenotypes:Influence on methadone plasma levels and response to treatment. Clin Pharmacol Ther.

[5] Crettol S, Déglon JJ, Besson J, Croquette-Krokkar M, Gothuey I, Hämmig R (2005). Methadone enantiomer plasma levels, CYP2B6, CYP2C19, and CYP2C9 genotypes, and response to treatment. Clin Pharmacol Ther.

[6] Fonseca F, de la Torre R, Díaz L, Pastor A, Cuyàs E, Pizarro N (2011). Contribution of cytochrome P450 and ABCB1 genetic variability on methadone pharmacokinetics, dose requirements, and response. PLoS One.

[7] Levran O, Peles E, Hamon S, Randesi M, Adelson M, Kreek MJ (2013). CYP2B6 SNPs are associated with methadone dose required for effective treatment of opioid addiction. Addict Biol.

[8] Ramli FF, Shuid AN, Mohamed P, Mohamed R, Sidik TA, Ikhwan TM (2019). Health-seeking behavior for erectile dysfunction in methadone maintenance treatment patients. Int J Environ Res Public Health.

[9] Ramli FF, Sidik TM, Mohamed IN (2020). Sexual inactivity in methadone maintenance treatment patients. Int J Environ Res Public Health.

[10] Wang SC, Ho IK, Tsou HH, Liu SW, Hsiao CF, Chen CH (2013). Functional genetic polymorphisms in CYP2C19 gene in relation to cardiac side effects and treatment dose in a methadone maintenance cohort. OMICS.

[11] Zahari Z, Lee CS, Ibrahim MA, Musa N, Yasin MA, Lee YY (2016). Relationship between ABCB1 polymorphisms and serum methadone concentration in patients undergoing methadone maintenance therapy (MMT). Am J Drug Alcohol Abuse.

[12] Bao YP, Liu ZM, Epstein DH, Du C, Shi J, Lu L (2009). A meta-analysis of retention in methadone maintenance by dose and dosing strategy. Am J Drug Alcohol Abuse.

[13] Chen CH, Wang SC, Tsou HH, Ho IK, Tian JN, Yu CJ (2011). Genetic polymorphisms in CYP3A4 are associated with withdrawal symptoms and adverse reactions in methadone maintenance patients. Pharmacogenomics.

[14] Coller JK, Barratt DT, Dahlen K, Loennechen MH, Somogyi AA (2006). ABCB1 genetic variability and methadone dosage requirements in opioid-dependent individuals. Clin Pharmacol Ther.

[15] de los Cobos JP, Sinol N, Trujols J, del Río E, Banuls E, Luquero E (2007). Association of CYP2D6 ultrarapid metabolizer genotype with deficient patient satisfaction regarding methadone maintenance treatment. Drug Alcohol Depend.

[16] Levran O, O'Hara K, Peles E, Li D, Barral S, Ray B (2008). ABCB1 (MDR1) genetic variants are associated with methadone doses required for effective treatment of heroin dependence. Hum Mol Genet.

[17] Shiran M, Chowdry J, Rostami-Hodjegan A, Ellis S, Lennard M, Iqbal M (2003). A discordance between cytochrome P450 2D6 genotype and phenotype in patients undergoing methadone maintenance treatment. Br J Clin Pharmacol.

[18] Wang SC, Tsou HH, Ho K, Lin KM, Liu YL (2013). Pharmacogenomics study in a Taiwanese methadone maintenance cohort. J Food Drug Anal.

[19] Chou R, Weimer MB, Dana T (2014). Methadone overdose and cardiac arrhythmia potential:Findings from a review of the evidence for an American pain society and college on problems of drug dependence clinical practice guideline. J Pain.

[20] Taheri F, Yaraghi A, Sabzghabaee AM, Moudi M, Eizadi-Mood N, Gheshlaghi F (2013). Methadone toxicity in a poisoning referral center. J Res Pharm Pract.

[21] Lin JH, Yamazaki M (2003). Role of P-glycoprotein in pharmacokinetics:Clinical Implications. Clin Pharmacokinet.

[22] L Mercer S, Coop A (2011). Opioid analgesics and P-glycoprotein efflux transporters:A potential systems-level contribution to analgesic tolerance. Curr Top Med Chem.

[23] Li Y, Kantelip JP, Gerritsen-van Schieveen P, Davani S (2008). Interindividual variability of methadone response. Mol Diagn Ther.

[24] Wang JS, Ruan Y, Taylor RM, Donovan JL, Markowitz JS, DeVane CL (2004). Brain penetration of methadone (R)-and (S)-enantiomers is greatly increased by P-glycoprotein deficiency in the blood-brain barrier of Abcb1a gene knockout mice. Psychopharmacology.

[25] Crettol S, Digon P, Golay KP, Brawand M, Eap CB (2007). *In vitro* P-glycoprotein-mediated transport of (R)-,(S)-,(R, S)-methadone, LAAM and their main metabolites. Pharmacology.

[26] Hassan HE, Myers AL, Coop A, Eddington ND (2009). Differential involvement of P-glycoprotein (ABCB1) in permeability, tissue distribution, and antinociceptive activity of methadone, buprenorphine, and diprenorphine:*In vitro* and *in vivo* evaluation. J Pharm Sci.

[27] Hung CC, Chiou MH, Teng YN, Hsieh YW, Huang CL, Lane HY (2013). Functional impact of ABCB1 variants on interactions between P-glycoprotein and methadone. PLoS One.

[28] Gibbs ME, Ledwitch K, Roberts A (2017). Probing the structural basis of P-glycoprotein transport of m-opioid receptor agonists:Methadone and loperamide. FASEB J.

[29] Gibbs ME, Wilt LA, Ledwitch KV, Roberts AG (2018). A conformationally gated model of methadone and loperamide transport by P-glycoprotein. J Pharm Sci.

[30] Hung CC, Chiou MH, Huang BH, Hsieh YW, Hsieh TJ, Huang CL (2011). EImpact of genetic polymorphisms in ABCB1, CYP2B6, OPRM1, ANKK1 and DRD2 genes on methadone therapy in Han Chinese patients. Pharmacogenomics.

[31] Gerber JG, Rhodes RJ, Gal J (2004). Stereoselective metabolism of methadone N-demethylation by cytochrome P4502B6 and 2C19. Chirality.

[32] Kharasch ED, Stubbert K (2013). Role of cytochrome P4502B6 in methadone metabolism and clearance. J Clin Pharmacol.

[33] Ansermot N, Albayrak Ö, Schläpfer J, Crettol S, Croquette-Krokar M, Bourquin M (2010). Substitution of (R, S)-methadone by (R)-methadone:Impact on QTc interval. Arch Intern Med.

[34] Bunten H, Liang WJ, Pounder D, Seneviratne C, Osselton MD (2011). CYP2B6 and OPRM1 gene variations predict methadone-related deaths. Addict Biol.

[35] Wang SC, Ho K, Tsou HH, Tian JN, Hsiao CF, Chen CH (2011). CYP2B6 polymorphisms influence the plasma concentration and clearance of the methadone S-enantiomer. J Clin Psychopharmacol.

[36] Totah RA, Allen KE, Sheffels P, Whittington D, Kharasch ED (2007). Enantiomeric metabolic interactions and stereoselective human methadone metabolism. J Pharmacol Exp Ther.

[37] van Dyk M, Marshall JC, Sorich MJ, Wood LS, Rowland A (2018). Assessment of inter-racial variability in CYP3A4 activity and inducibility among healthy adult males of Caucasian and South Asian ancestries. Eur J Clin Pharmacol.

[38] Foster DJ, Somogyi AA, Bochner F (1999). Methadone N-demethylation in human liver microsomes:Lack of stereoselectivity and involvement of CYP3A4. Br J Clin Pharmacol.

[39] Wu D, Otton S, Sproule B, Busto U, Inaba T, Kalow W (1993). Inhibition of human cytochrome P450 2D6 (CYP2D6) by methadone. Br J Clin Pharmacol.

[40] Lin E, Lin CH, Lane HY (2020). Precision psychiatry applications with pharmacogenomics:Artificial intelligence and machine learning approaches. Int J Mol Sci.

